# Salmagundi

**Published:** 1884-01

**Authors:** 


### SALMAGUNDI.

EDITED BY E. G. BETTY, D.D.S.

### INTRODUCTORY.

It is eminently proper that at the beginning of the new year, every one should form new resolutions for his or her conduct, and clinch the matter by turning over a new leaf, and following them to the letter.

So, too, will the REGISTER, "the father of them all," the oldest in continuous service, in this the thirty-eighth year of its useful life, form new resolutions, and actually turn over a new leaf, and call it *Salmagundi*, wherein all are bade a hearty welcome and where their offerings at the throne of knowledge may be duly received, *acknowledged*, and recorded.

Our object is to establish a department for the introduction of choice and interesting extracts from the exchange list, that would not otherwise reach those whom they will benefit; to offer a medium through which the readers of the REGISTER may confer with one another by question and answer; to encourage those who *could* write, if they tried their hands at it, to communicate in brief, methods of practice, and little things of interest and practical importance that are not generally known; to collect items about societies, and, in fact, any or all of the countless odds and ends that, like the modest violet, hide their heads and pine away in some obscure corner for want of light and ventilation.

So, gentle reader, "chip" in, and help us with a will; look among the papers in your desk; dig into your note-books; scour your diaries; and give to us for your professional brethren, the little tid-bits that taste so good and whet our appetites for that which is more substantial, though, possibly, less digestible.

All communications, extracts, etc., for this department, should be addressed to E. G. BETTY, D.D.S., care Messrs. SPENCER & CROCKER, Cincinnati, Ohio.

### "SCIENTIFIC"—ETYMOLOGICAL.

*Sci*, sigh, "to inhale a larger quantity of air (wind) than usual and immediately expel it," as he does "who adequatistically presents us with a statumentary attestination of the progresses by which the inaudible sound of an unheard noise becomes cognomystical music in all its derivitivities in sounds, with predominationaries and subdoministitudes of mineral, vegetable, animal, human, and assinine characteristicisms."

*En*, en, "a prefix to many *En*-glish words," also "a termination," as, for instance "gold-*en* pipp-*en*; it also occurrs near the middle of many words, as in "nasc-*en*-t."

*Tif*, tiff, "liquor, or rather a small draught of liquor. German beer, maybe, as sometimes seen in a "saloon style of," etc., also "to be in a pet, *rare*," at least we hope so.

*Ic*, hic, an abbreviation of "hic-cough, a spasmodic inspiration," as in inhaling that large quantity of wind in "sigh." And now we have the derivitivities of the several parts of the word *Sigh-en-tiff-hic*, which in its entireteristity means "evincing profound and systematic knowledge; as a scientific arrangement of *fossils*," (italics ours) bone phosphate, nascent energy, etc.

And to conclude the matter:

"The Devil and the Dutch and the Dun Cow fit,
The Devil whipped the Dutch and the Dun Cow quit."

Watt a good thing for them that they did, for they might have been arrested, hauled up before the Mayr and fined.

### UNIFORM TYPE.

The quaint Isaac D'Israeli tells us that "Ancillon was a great collector of curious books, and dexterously defended himself when accused of the *Bibliomania.* He gave a good reason for buying the most elegant editions; which he did not consider merely as a literary luxury. He said the less the eyes are fatigued in reading a work, the more liberty the mind feels to judge of it; and as we perceive more clearly the excellencies and defects of a printed book than when in MS., so we see them more plainly in good paper and clear type than when the impression and paper are both bad." So the REGISTER will take the hint and print its *Salmagundi* in type uniform with the rest, thus relieving the tired eyes of its readers and the better to enable them to perceive its "excellencies," for we don't intend to have any "defects" "if the Court know herself, and she think she do."

"THEY fit an' fit,
And gouged and bit,
Until the ground
For miles around,
Was kivered, with thar blood."

For the particulars of this desperate encounter, second only to the great battle in the early Dutch settlements of New York, so ably described by Washington Irving, in "Knickerbocker," the reader is kindly referred to page 589 in the December number of the *Ohio State Journal.*

### COPPER WEDGE AND MATRIX.

CINCINNATI, *December* 22, 1883.

DEAR DR. BETTY: Did it ever occur to you as you passed a penny to the newsboy for an evening paper, that the world had mined during the present century 3,000,000 tons of copper; and that our own country produced last year 30,000 tons? What becomes of it all? I might enumerate a few of the many articles into whose composition it largely enters, but will only mention the fact that it is included in the making of many useful and valuable articles all the way from a pin to a Tyler-Davidson fountain. We make use of it in dentistry for alloying metals, and it has been used in the form of amalgam for filling teeth. I use it in the form of a wedge for separating the teeth when filling, with great satisfaction.

Get a piece of copper wire, size 16 of the gauge-plate, three inches in length; fasten in a pin-vise and with a file taper from the center to each end, rotating it meanwhile to make it round, bringing the end to almost a sharp point; finish with emery cloth; finally annealing in the spirit flame. It may be used advantageously between all the teeth with the possible exception of the molars. To apply (after having adjusted the rubber dam) introduce a thin spatula between the teeth to be separated until all space possible is obtained, then insert the wedge above the spatula and near the gum, pushing through with the finger as far as possible. By bending the wedge back against the lingual surfaces of the teeth, loosening will be prevented; the spatula may now be removed, and the outside portion of the wedge also bent aside, thus serving to keep the rubber out of the way. I have obtained sufficient space in this way that would otherwise require days of time, and much inconvenience to the patient, in a few minutes, and with very little pain to the patient. After using several times the wedge will need annealing again. Another use I have made of copper is in the form of a matrix for the insertion of *plastic* fillings.

Take a piece of wire as thick again as the above, and about half an inch long; lay it transversely on the round horn of a small anvil, and, with a hammer, strike it in the center until it becomes flattened to such a thickness as may be desirable, usually the thickness of ordinary gold plate. You will then have an oval wedge and matrix combined. When inserted between the teeth, the ends being thick, will cause the matrix to adjust itself nicely to the shape of the tooth. In some cases this matrix will be useful when inserting a gold filling.

N. S. Hoff, D.D.S.

Mutilations of the Teeth.—Ethnographers who have minutely described mutilations of the teeth in other parts of the world have said nothing of a similar practice among the natives of the two Americas. The practice is not common in the Western World; and it is a little singular that people who deform to an extraordinary degree their lips, noses, cheeks, and ears, respect the integrity of their teeth. The historians of this practice overlook the abrasions noticed by Vancouver among the Indians of Trinidad Bay, and by Petitot among the Tchiglits at the mouth of the Mackenzie and of the Anderson. Finally, no notice is taken of dental mutilations formerly in use in Mexico and Yucacan, upon which Sahagun, Landa, and Mota Padilla, have furnished information. M. Hamy has gathered from the last-named writers their allusions to these subjects, and prepared an illustrated monograph. The drawings indicate both filing and perforations.—*Bull. soc. anthrop. Paris, v.* 879.

### REX MAGNUS.

A writer in *Science* made an examination of the "viandine" brand of the new preservative "Rex magnus," and finds that it is composed of

| | |
|---|---|
| Boracic acid ............................ | 67 per cent. (with Borax) |
| Borax .................................... | |
| Potassic chloride ........................ | 15 " " |
| Water .................................... | 18 " " |

A series of experiments with it demonstrated that it would preserve meats and fish for some length of time, even in a warm temperature, if, like a steak, the surface is much greater than the thickness, though with a slight deterioration of the flavor.

Whether this preparation could be used for dental purposes or not can only be decided by trial.

Carbolic Acid.—From the results of a series of experiments, W. Meyke arrives at the following conclusions: 1. Pure carbolic acid should be colorless, have the proper boiling point, and be entirely volatalized by heat. 2. The congealing point is of secondary importance. 3. Carbolic acid is colored red when kept in glass vessels containing lead. 4. The best vessels for keeping carbolic acid are made of tinned sheet iron.—*Phar. Zeit. Russl.*

VARNISHES MADE WITH BORAX.—It is well known that shellac dissolves in borax solution, and this solution is often utilized for various purposes, both as varnish and cement. The following are the proportions employed: Ten parts of borax, thirty parts of coarsely pulverized shellac, and two hundred of water. It is dissolved by warming on a steam bath for a few hours. When cold it may be filtered. To make it more pliable add a few drops of glycerine. It may be given various colors by introducing soluble pigments.—*Scientific American.*

M. PASTEUR has proved that the burial of diseased animals does not destroy the germs of disease, or obviate the chance of infection to any animals who may afterwards feed on the ground above where the body of the diseased animal was buried.—*Science.*

